# Investigating the role of bacterial raw milk community members in chlorate reduction

**DOI:** 10.1099/acmi.0.001088.v3

**Published:** 2026-04-20

**Authors:** Meghana Srinivas, Orla O'Sullivan, Paul D. Cotter, Douwe van Sinderen, John G. Kenny

**Affiliations:** 1 Teagasc Food Research Centre, Fermoy, Co. Cork, Ireland; 2 School of Microbiology, University College Cork, Cork, Ireland; 3 APC Microbiome Ireland, University College Cork, Cork, Ireland; 4 VistaMilk Research Ireland Centre, Cork, Ireland

**Keywords:** chlorate, chlorate-reducing bacteria, dairy, food safety, milk microbiome, shotgun metagenomic sequencing

## Abstract

Chlorine-based detergents, used in the dairy industry for cleaning, often degrade into chlorate, contaminating milk and dairy products. Consumption of chlorate has been linked to thyroid dysfunction in adults and impaired neurological development in infants. Despite the ban on chlorine-based detergents in Ireland since 2021, chlorate contamination remains a problem in the dairy supply chain. A recent study identified chlorate-reducing bacteria naturally present in raw milk, highlighting their potential for mitigating chlorate. In this study, shotgun metagenomic sequencing was applied to determine the effects of chlorate concentration and incubation conditions on the raw milk microbiome, specifically focusing on chlorate-reducing bacteria within the community. Chlorate-spiked milk samples from different farms showed reductions in chlorate levels over time, from day 10 onwards when stored at 4 °C and after 24 h when incubated at 25 °C. *Pseudomonas* and *Lactococcus* were observed as the most dominant taxa in raw milk samples stored at 4 °C and 25 °C, respectively. High abundances of *ydeP* and *narG* genes were observed for 4 °C samples and were attributed to *Pseudomonas* and various low-abundance genera, respectively. High abundances of the *napA* gene were noted in 25 °C samples and were attributed to the *Lactococcus* genus. Overall, this study highlights the presence of naturally occurring chlorate-reducing bacteria as part of the raw milk microbiome and identifies multiple genes linked to various pathways potentially involved in chlorate reduction. Furthermore, incomplete pathways potentially involved in chlorate reduction were found, suggesting metabolic cross-feeding and underscoring the community roles bacteria play in chlorate reduction in raw milk. Additionally, a few previously uncharacterized genes, such as *ydeP*, belonging to the DMSO reductase gene family were identified at high abundances in samples that showed chlorate reduction, emphasizing the need for further biochemical characterization of these genes to better understand the pathways involved in chlorate reduction in milk.

## Introduction

Chlorate is a chemical residue of concern in the dairy supply chain. It has been identified as having an inhibitory effect on thyroid hormone synthesis, which can disrupt thyroid function in adults and affect neurological development in infants and young children [1,4]. Given that the diets of infants and young children can largely consist of infant milk formula and other dairy products, they are at a high risk of chlorate exposure and its associated detrimental effects [5,8].

Chlorate is formed by the unavoidable decomposition of hypochlorite in concentrated sanitation solutions. When these decomposed hypochlorite solutions are used to clean equipment and contact surfaces at farms and food processing facilities, chlorate residues can inadvertently be introduced into milk and milk products [9,10]. This contamination poses potential health risks to consumers. Therefore, it is necessary to monitor chlorate levels in milk, with the European Union (EU) setting a maximum residue limit (MRL) of 0.10 mg/kg, or 100 parts per billion (ppb), for all milk in its ready-to-use state [11]. Over 1.7 million tonnes of milk is exported from Ireland to global markets, and nearly 13% of the world’s infant milk formula is produced in the country [12]. As a result, controlling chlorate contamination is particularly important for the Irish dairy industry. To address this potential health risk, Ireland has prohibited the use of chlorine-based detergents and sanitizers on farms and in processing plants as of 1 January 2021 [13]. Instead, ‘chlorine-free’ alternatives, such as sodium hydroxide detergents, phosphoric/nitric acid descalers and peracetic acid disinfectants, are being used [14,17]. However, despite these measures, low levels of chlorate continue to be detected in milk and dairy products, which have been attributed to the use of chlorinated water in cleaning processes [13,15]. This ongoing issue underscores the need for stringent monitoring and regulation of chlorate levels in dairy production to ensure safety and quality.

Chlorate removal through chemical methods, often involving filtration, has been investigated. However, these methods need to be optimized further before their application on a large scale [18]. Microbial-driven chlorate reduction has been extensively employed for soil and wastewater bioremediation and could provide a useful solution to the chlorate residue problem in the dairy industry [19,21]. Although the potential contribution of microbes to reduce chlorate levels in the dairy industry is under-explored, one study on raw milk samples reported a significant decrease in chlorate levels when chlorate-spiked milk samples had been stored at 4 °C for 10 days or more, particularly in raw milk that had not been ultra-high temperature-treated or pasteurized [22]. This suggests that raw milk naturally contains bacteria capable of reducing chlorate, indicating an alternative, microbiological means of chlorate removal in milk. Chlorate-reducing bacteria, or dissimilatory (per)chlorate-reducing bacteria (DPRB), when they are capable of reducing both perchlorate and chlorate, have been isolated and identified from various environmental sources, including soil, wastewater and aquatic sediments [19,20, 23,25]. DPRB reduces perchlorate to chlorate and then chlorate to chlorite using perchlorate reductase (PcrA). The resulting chlorite is then converted to chloride ions and molecular oxygen via a dismutation reaction catalysed by chlorite dismutase (Cld). In this process, perchlorate and chlorate are reduced to harmless chloride ions and oxygen by DPRB [19,24]. Some studies have indicated that certain chlorate-reducing bacteria possess enzymes, specifically chlorate reductase (ClrA), which only reduce chlorate and not perchlorate [26,28]. Both PcrA and ClrA belong to the dimethyl sulfoxide (DMSO) family of enzymes, which also includes nitrate reductases, selenate reductases, trimethylamine N-oxide (TMAO) reductases and biotin sulfoxide reductases [19,29]. These enzymes have also been reported to be involved in chlorate reduction [29,34]. Specifically, the genes encoding membrane-bound nitrate reductases, *narGHI* in some species, have commonly been implicated in chlorate reduction, sometimes even performing the latter reaction at higher rates than nitrate reduction [21,33, 35,39]. The only existing study to isolate chlorate-reducing bacteria from milk suggested that the nitrate reductase NarGHI produced by *Hafnia* species was involved in chlorate reduction [22]. While other studies have investigated the chlorate reduction capabilities of components of the rumen microbiome and members of the *Bifidobacterium* genus that dominate the infant gut microbiota, the metabolic pathways employed by these microbes to reduce chlorate have not been explained [40,41].

In general, while existing studies have highlighted the potential of milk substrates to harbour chlorate-reducing bacteria, the specific pathways utilized by these bacteria need to be better understood. Understanding such pathways may facilitate the application of bacteria for chlorate mitigation in the dairy industry. In the current study, shotgun metagenomic sequencing was applied to analyse the microbial composition of raw milk samples collected from farms across Ireland, aimed at identifying bacterial species capable of chlorate reduction. Additionally, the effects of incubation conditions and chlorate concentrations on raw milk samples were evaluated to determine optimal conditions for chlorate reduction and to reveal genes that may be responsible for this process.

## Methods

### Initial screening for the selection of incubation conditions

Suitable chlorate concentrations and incubation conditions were determined through an initial screening, wherein conditions showing reductions in chlorate concentration were selected for further analysis. The incubation condition of 4 °C for 14 days, with 100 ppb of chlorate added to raw milk, achieving a final concentration of 1.2 mM, was chosen based on the methodology used for initial screening described by McCarthy *et al*. [22]. This chlorate concentration is also biologically relevant, aligning with the MRL set by the EU for ready-to-use milk. Moreover, a reduction in chlorate levels was observed from day 10 onwards in the chlorate-spiked samples incubated at 4 °C [22]. Therefore, this timepoint was selected for further analysis, including chlorate residue and metagenomic analysis in the present study. For the 25 °C incubations, a range of chlorate concentrations was tested on raw milk samples in duplicates, ranging from 250 to 120,000 ppb. Among these, 16,000 ppb or 16 parts per million (ppm) was identified as the highest chlorate concentration at which a reduction in chlorate level was observed following 24 h of incubation at 25 °C (Table S1, available in the online Supplementary Material) and, therefore, was selected for further analysis.

### Sample collection, preparation and chlorate spiking

**Fig. 1.**
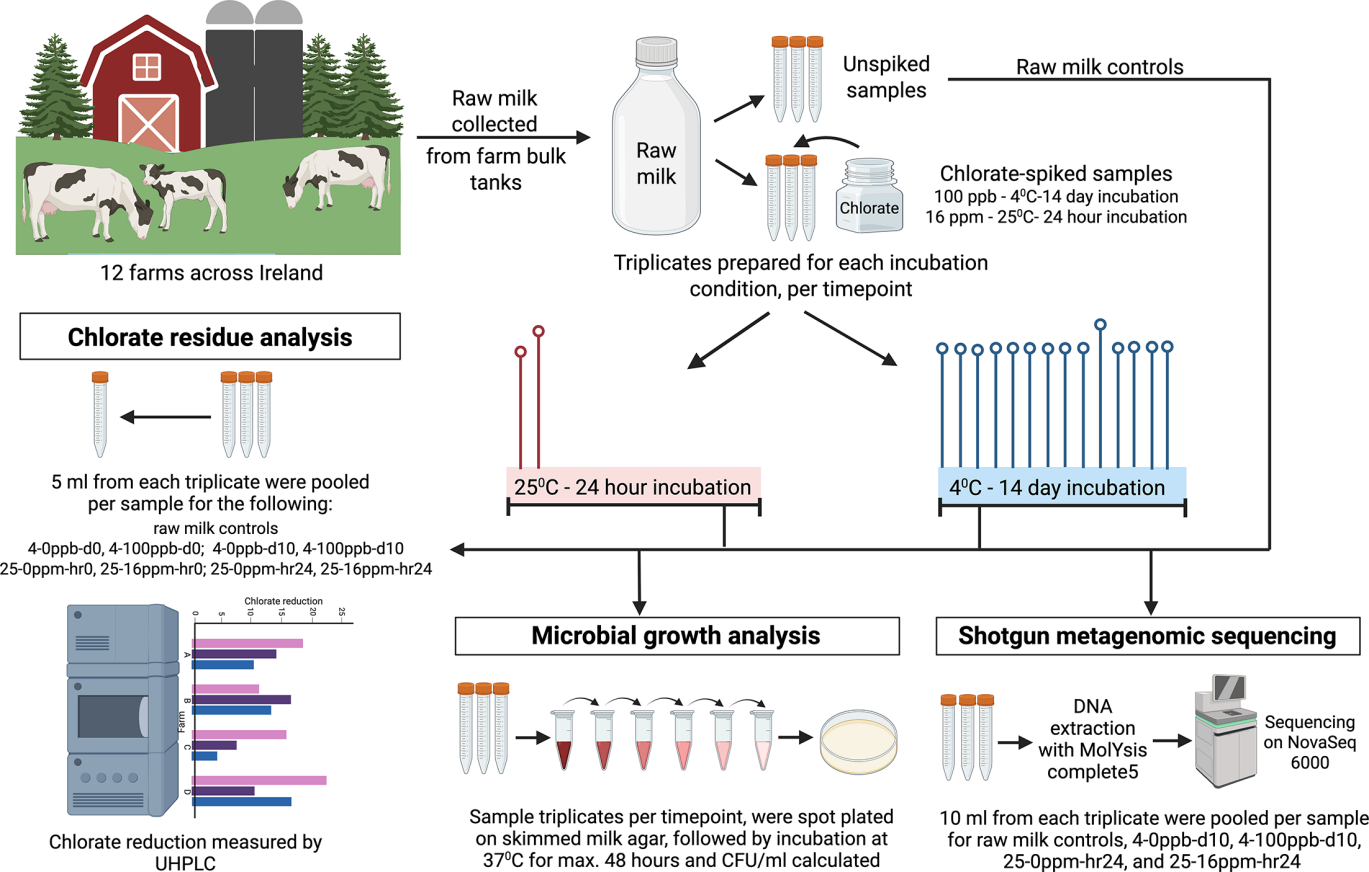
Overview of study design. Schematic representation of the study design showing sample collection, processing and analysis methods applied (created with BioRender.com).

Raw bovine milk samples (3 l) were collected from the bulk tanks of 12 farms across Ireland during August and September 2022. The duration between sample collection, transport and processing was 48 h at maximum, with transport and storage until processing at 4 °C, mimicking the raw milk storage conditions on farm bulk tanks. Raw milk samples (20 ml) from each farm were incubated in triplicate, with and without added chlorate (Chlorate IC standard supplied by Merck), under different incubation conditions. The raw milk samples, as is and through the incubation conditions, i.e. at time point 0 and at the end of incubation for (i) 14 days at 4 °C and (ii) 24 h at 25 °C, underwent chlorate residue analysis (as described below) for chlorate-spiked and -unspiked samples. Raw milk and chlorate unspiked samples consistently showed chlorate concentrations below the detection limit of 2 ppb (Table S1) and, therefore, for this study are henceforth referred to as the raw milk controls (for the raw milk samples) and the 0 ppm samples (for the chlorate unspiked samples). Overall, the samples included 0 ppb and 100 ppb at 4 °C for 14 days, 0 ppm and 16 ppm at 25 °C for 24 h and the raw milk controls collected from the bulk tank of each farm that did not undergo incubation under either 4 °C for 14 days or 25 °C for 24 h and did not have any chlorate added to them. The raw milk controls are labelled in the format of farm number-control for each farm. A schematic of the workflow used to collect, process and analyse the raw milk samples is presented in Fig. 1.

### Microbial growth analysis

Microbiological growth was assessed for the triplicates for each sample using plate counts. This was performed using a spot plate method with plating onto skimmed milk agar (Sigma), followed by incubation at 37 °C for a maximum of 48 h and colony counts calculated in c.f.u. ml^−1^. Timepoints for microbiological growth assessment were taken every 24 h for the 4 °C incubated samples for 14 days and at hour 0 and hour 24 for the 25 °C incubated samples. From here on, samples are referred to using their abbreviated forms following a general format of incubation temperature–chlorate concentration–incubation timepoint, wherein incubation timepoint is abbreviated as ‘d’ for days for the 4 °C samples and ‘hr’ for hours for the 25 °C samples (e.g. 4-0ppb-d10 stands for an unspiked sample at day 10 of incubation at 4 °C). Where farm-specific samples are mentioned, the general format of farm number–incubation temperature–chlorate concentration–incubation timepoint has been applied.

### Chlorate residue analysis

Chlorate residue analysis was performed on the following: raw milk controls, 4-0ppb-d0, 4-100ppb-d0, 4-0ppb-d10, 4-100ppb-d10, 25-0ppm-hr0, 25-16ppm-hr0, 25-0ppm-hr24 and 25-16ppm-hr24 samples. Triplicates prepared per sample were pooled together using 5 ml per replicate, making a total volume of 15 ml used for chlorate residue analysis. The quantification of chlorate, as well as perchlorate, ions was performed by ultra-high-performance liquid chromatography coupled to MS/MS. Residue analysis was performed by FBA labs in Waterford, Ireland, following the standard protocol described by Moloney *et al*. [42] using a proprietary ISO17025 accredited method [43] that has been developed and validated specifically for the detection of chlorate and perchlorate residues in milk and milk powders. The lowest detection limit for chlorate and perchlorate residues was 0.0020 mg kg^−1^, or 2 ppb, for milk samples.

### Sample processing and DNA extraction

Samples were stored at −80 °C until pre-processing. A total of 60 samples, including raw milk controls, 4-0ppb-d10, 4-100ppb-d10, 25-0ppb-hr24 and 25-16ppb-hr24 samples, were processed prior to DNA extraction as per a previous study with a few minor modifications [44]. Sample triplicates from each timepoint and incubation condition were centrifuged at 4,500 ***g*** for 20 min at 4 °C. After centrifugation, cream and supernatant were discarded, and pellets were resuspended in 800 µl of sterile PBS (Merck), and the triplicates were pooled. These pooled pellets were subjected to two washing steps using sterile PBS and centrifuged at 13,000 ***g*** for 1 min, following which the supernatant was discarded, as per Yap *et al*. [44]. The pellet was stored at −20 °C until DNA extraction. Of the 60 samples processed, 2 samples (F4-4-0ppb-d10 and F6-4-0ppb-d10) were curdled and so underwent an initial and additional centrifugation step at 350 ***g*** for 10 min at 4 °C, following which supernatants were collected and processed as described above. DNA extraction was performed as per the manufacturer’s instructions using the MolYsis complete5 kit (Molzym GmbH and Co. KG, Bremen, Germany), which performs host DNA removal, thereby improving microbial community coverage during sequencing. A total of 30 µl of genomic DNA (gDNA) was eluted into deionized water for downstream sequencing and stored at −20 °C prior to library preparation. All gDNA quantifications were performed using the Qubit dsDNA HS assay kit (Invitrogen) as per the manufacturer’s guidelines.

### Library preparation and sequencing

The 60 gDNA-extracted samples were prepared for shotgun metagenomic sequencing using the Illumina DNA Prep kit according to the Illumina DNA prep reference guide (Illumina, CA, USA), with multiplexing performed using unique dual indexes from the IDT Set 1 of barcodes (Integrated DNA Technologies, Coralville, IA, USA). To minimize overamplification while maximizing the input available for sequencing from low-concentration samples, samples were divided into three gDNA input categories based on the amount of gDNA arising from DNA extraction. Samples with less than 0.5 ng µl^−1^ gDNA concentration were brought to a standard input of 2 ng in 30 µl and underwent 12 PCR cycles. Samples with more than 0.5 ng µl^−1^ but less than 2 ng µl^−1^ gDNA concentration were made to a standard input of 10 ng in 30 µl and underwent eight PCR cycles. All samples with more than 2 ng µl^−1^ gDNA concentration were brought to a standard 50 ng in 30 µl input and underwent five PCR cycles. PCR conditions were followed according to the Illumina DNA prep reference guide. The indexed and cleaned libraries of all samples were pooled to an equimolar concentration of 10 nM. The pooled library was checked on a 2100 Bioanalyzer instrument (Agilent Technology, CA, USA), using the Agilent DNA 1000 Kit. Following this, the pooled library was sequenced on the Illumina NovaSeq 6000 sequencing system (2×150 bp) using an S2 flowcell at the Teagasc DNA Sequencing Facility.

### Sequence analysis

All analysis tools were used with default parameters unless specified otherwise. Quality checks were performed using FastQC [45] (version 0.11.8) and MultiQC [46] (version 1.9). Quality trimming and adapter removal were carried out using Trim Galore [47] (version 0.6.1). Host reads were aligned to the bovine genome (*Bos taurus*) and removed with Bowtie2 [48] (version 2.4.4), and the number of reads obtained per sample and reads lost post-host removal are provided in Table S2. Insufficient sequencing data were obtained for six samples, F6-control, F7-control, F12-control, F10-4-100ppb-d10, F3-25-16ppm-hr24 and F4-25-0ppm-hr24, and these were excluded from downstream analyses. In the case of the raw milk controls, this was likely due to the very low amount of microbial material present at T0. The low yields for the latter three samples possibly result from contaminants interfering with library preparation. The quality-controlled and host-removed short reads, hereafter referred to as short reads, were assigned taxonomic profiles using Kraken2 [49] (version 2.1.1) with the Genome Taxonomy Database (GTDB) version R08-RS214) [50], containing Bacteria and Archaea. As differences in the read numbers per sample were observed, a rarefaction analysis was conducted to determine the potential impact of this variation on downstream analysis outcomes. For this, the short reads were rarefied to one million reads per sample with ten iterations, as one million was the lowest number of reads obtained for a sample. Reads were rarefied using seqtk [51] (version 1.3) with seeds, 5, 11, 20, 45, 62, 75, 90, 135, 150 and 189 provided for each of the 10 iterations. Each of the subsample iterations was compared with the other and with their non-rarefied counterparts to determine the extent of variation introduced within a sample due to differences in their sequencing depth. However, no significant differences were observed between subsamples and against their non-rarefied counterparts (Figure S1). Nonpareil [52] (version 3.4.1) was used to estimate microbial diversity coverage and sequencing redundancy (Figure S2). In agreement with rarefaction analysis, most samples reached redundancy at approximately one million reads. Two control samples (F1-control and F11-control) showed reduced redundancy, likely due to high host-read content within the raw milk samples. As no significant differences were observed between rarefied and non-rarefied datasets, non-rarefied reads were retained for all downstream analyses.

### Construction of metagenome-assembled genomes

The MetaWRAP [53] (version 1.3.2) pipeline was used to assemble metagenome-assembled genomes (MAGs) from the quality-trimmed and host-removed reads. Contig assembly was performed using MEGAHIT within the assembly module. Assembled contigs were binned with metabat2, maxbin2 and concoct options of the binning module. Bins were then refined and reassembled using the bin_refinement and reassemble_bins module. Bins were quality checked using CheckM2 [54] (version 0.1.3). High-quality MAGs, with ≥90% completeness and <5% contamination, were retained for downstream analyses. Taxonomic classification of the MAGs was performed using GTDB-tk [55] (version 1.3.0). For two samples, F11-control and F9-4-100ppb-d10, having 149 and 496 million reads, respectively, the quality-trimmed and host-removed reads were first deduplicated using BBMap [56] (version 38.22) clumpify.sh with the dedupe option before MAG assembly with the MetaWRAP pipeline.

### Identification of genes with chlorate reduction potential

The eggNOG-mapper v2 [57] (version 2.1.7) was employed to annotate genes within the obtained MAGs, utilizing the diamond aligner [58], and Prodigal [59] to predict ORFs. These predicted genes were subsequently screened for those potentially involved in chlorate reduction, including perchlorate reductase (*pcrA*), chlorate reductase (*clrA*), nitrate reductases in the DNR and denitrification pathways (*narG*, *narH*, *narI*, *napA* and *napB*) [33], nitrate reductase in the ANR pathway (*nasA*) [35], selenate reductases (*ynfE* and *ynfF*) and DMSO reductases (*dmsA2* and *dmsA*) [29,34], TMAO reductases (*torA* and *torZ*) [30,32], biotin sulfoxide reductase (*bisC*) [31,60] and chlorite dismutase Pfam 06778 (*cld*) [35], along with one formate-dehydrogenase like molybdopterin oxidoreductase (*ydeP*), another uncharacterized molybdopterin oxidoreductase (labelled as *unk_mol*) and other members identified as belonging to the chlorite dismutase family Pfam 06778 (*ywfI*). Additionally, genes involved in nitrite, nitric oxide and nitrous oxide reduction within the dissimilatory nitrate reduction (DNR) and denitrification pathways were also screened. Protein sequences of the genes of interest were extracted from the MAGs and clustered at 98% identity using CD-HIT [61] (version 4.8.1). A diamond database was created from the resulting 98% clustered predicted genes, consisting of 232 protein sequences. Short reads were first merged into one file, using IDBA [62] (version 1.1.3), fq2fa with the merge option, and then mapped against the above database using diamond [58] (version 2.1.8–162) with the blastx option to identify potential chlorate reduction genes within the short-read dataset. An identity threshold of ≥95% was applied to the resulting matches, and those passing this threshold were used to evaluate gene abundances per sample and to perform differential abundance analysis and Spearman correlation, as explained in the section below.

### Statistical analysis and data visualization

Statistical analysis and data visualization were performed in R (version 4.1.2). Alpha diversity metrics, i.e. observed taxa (index=‘richness’), Shannon index (index=‘shannon’) and Simpson diversity (index=‘simpson’, which reports 1-D values), for the short-read datasets were calculated using the vegan package [63] (version 2.6.4), and the corresponding statistical tests applied were Kruskal–Wallis, followed by pairwise Wilcox test with p-adjustment using Benjamini–Hochberg method using the stats (version 4.1.2) package. Beta diversity calculations for the short-read datasets were performed using the Bray–Curtis method, along with the corresponding statistical tests of PERMANOVA (permutational analysis of variance) and pairwise PERMANOVA specifying 10,000 permutations, which was performed using the adonis function from the vegan package. A relative abundance threshold of 0.001% at the species level was used when calculating alpha and beta diversity metrics. ALDEx2 [64] (version 1.26.0) was used to perform differential abundance analysis for the genes of interest across various incubation conditions and chlorate reduction categories. This analysis was conducted using the ALDEx (for two categories) and aldex.glm modules (for more than two categories), with the Benjamini–Hochberg method applied for false discovery rate (FDR) control. The rcorr function in the Hmisc [65] (version 5.0–1) package was used to perform Spearman correlations between genes of interest and incubation conditions and chlorate reduction categories. For all statistical tests, significance was accepted as *P*<0.05 and FDR<0.05. Data visualization was performed using ggplot2 [66] (version 3.4.2) for most plots, except ComplexHeatmap [67] (version 2.10.0), which was used to visualize the differential abundance outputs of ALDEx2 and corrplot (version 0.92) for Spearman correlation.

## Results

### Microbes innate to raw milk are capable of reducing chlorate

**Fig. 2.**
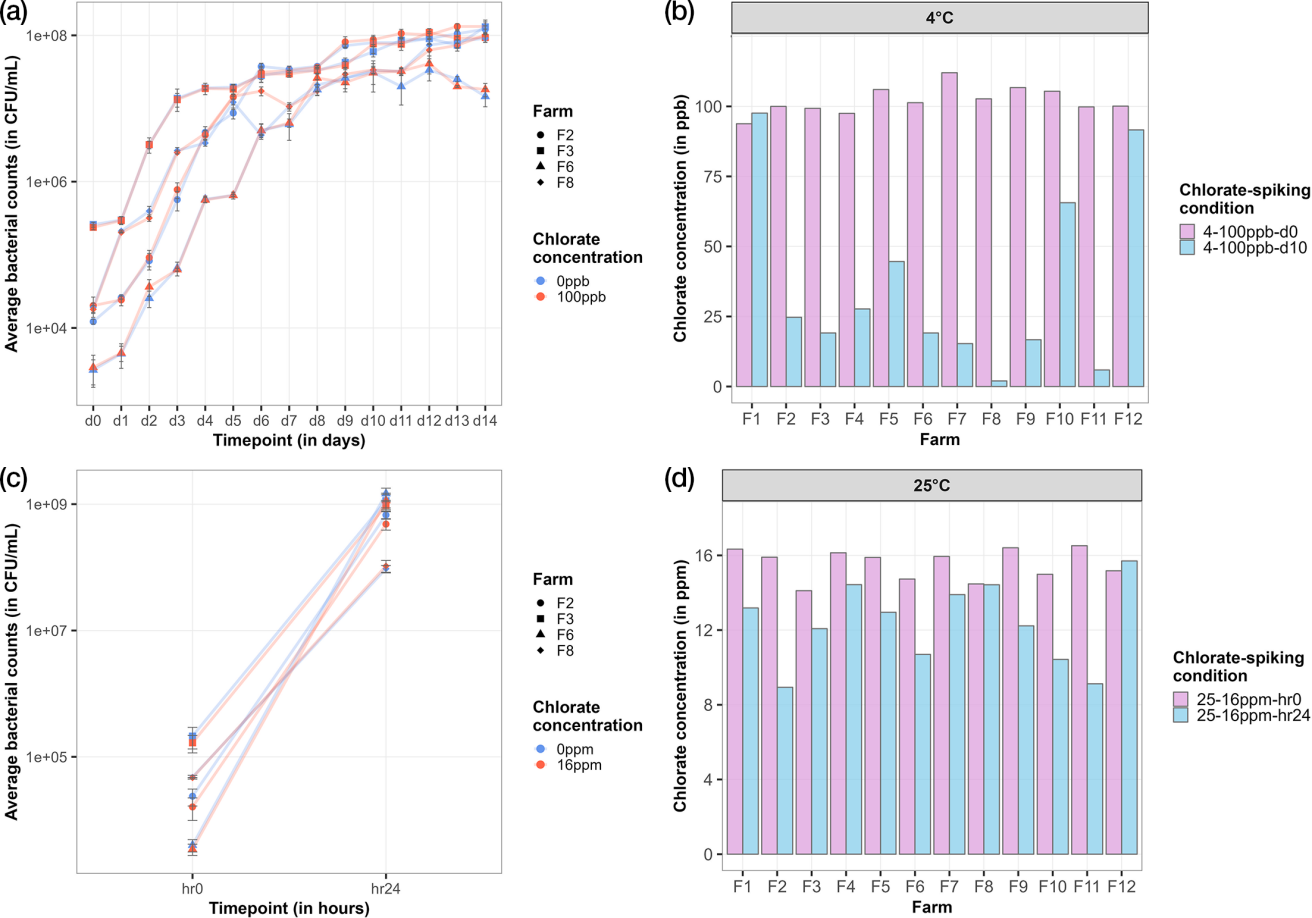
Microbial growth curves at 4 °C and 25 °C incubation (left panels). Bacterial growth curves showing mean counts (c.f.u. ml^−1^) on the *y*-axis over time on the *x*-axis in both chlorate-spiked (red) and unspiked (blue) samples following incubation at (**a**) 4 °C for 14 days and (**c**) 25 °C for 24 h. Triplicate c.f.u. ml^−1^ counts were averaged per farm, timepoint and chlorate-spiking condition, represented by each point. Error bars represent standard deviation. Four farm samples representative of the trends observed across the 12 farms are displayed. Chlorate residue concentrations at 4 °C and 25 °C incubation (right panels). Bar plots indicating chlorate concentrations (*y*-axis) for chlorate-spiked samples with incubation at (**b**) 4 °C for 14 days and (**d**) 25 °C for 24 h per farm (*x*-axis). Pink bars represent chlorate concentration at time point 0, while blue bars show chlorate concentration at the end of incubation. Farms were treated as independent biological replicates (*n*=12), where growth curve and chlorate residue measurements obtained before and after incubation were paired by farm.

The ability of microbes that are naturally present within raw milk to reduce chlorate was tested under the two incubation conditions, 4 °C for 14 days and 25 °C for 24 h. The average bacterial load was calculated in c.f.u. ml^−1^ for microbial growth analysis, across the timepoints, for each incubation condition. Reduction of chlorate residues in 4-100ppb-d10 and 25-16ppm-hr24 samples (labelled in the format of farm number-incubation temperature–chlorate concentration–incubation timepoint) was measured. Bacterial counts consistently increased over time across all incubation conditions, regardless of chlorate levels in the samples. For both incubation conditions, no difference in microbial levels was observed between chlorate-spiked and unspiked samples, despite the presence of chlorate at 100 ppb and 16 ppm in the 4 °C and 25 °C spiked samples, respectively (Fig. 2, panels a and c for four representative farms, Figure S3 for all 12 farms). Upon incubation, the bacterial load increased by 4 log-fold, to 10^8^ c.f.u. ml^−1^, in the 4-0ppb-d14 and 4-100ppb-d14 samples, and by 5 log-fold, to 10^9^ c.f.u. ml^−1^, in the 25-0ppb-hr24 and 25-16ppb-hr24 samples. Reduction in chlorate residues (>66%) was observed in most 4-100ppb-d10 samples (Fig. 2b). Although the increase in bacterial load was uniform across all samples and incubation conditions, the extent of chlorate reduction varied, potentially attributed to differences in microbial composition across the sample types. Among the samples showing high chlorate reduction, F8 exhibited the greatest chlorate reduction potential, reducing chlorate to below detection limit (<2 ppb) post-incubation. For the 25-16ppm-hr24 samples, generally either low (<33%) or medium (33%–66%) levels of chlorate reduction were observed. Notably, samples from farms F2 and F11 consistently reduced large amounts of chlorate across the two incubation conditions, with high chlorate reduction capacity at 4 °C and medium capacity at 25 °C.

### The microbial composition of raw milk was affected more by incubation temperature than by chlorate presence

Shotgun metagenomic sequencing was employed to ascertain differences in microbial composition among the spiked and unspiked samples under the two incubation conditions, as well as with the raw milk controls. The raw milk controls were sourced from 12 farms across Ireland. However, for three farms (F6, F7 and F12), inadequate sequencing data were obtained, likely due to the very low amount of input material available from these raw milk controls, preventing the determination of their raw milk microbiomes. Among the nine raw milk controls, a total of 3,423 unique genera and 20,596 unique species were identified. Many species identified were present at low relative abundances, i.e. below 1.5%, contributing between 16.08% (F3-control) at the lowest end to as high as 93.21% (F4-control) of the raw milk control’s microbiome, varying per farm. In total, 70 genera and 210 species were found across the raw milk control samples when no relative abundance threshold was applied. Raw milk microbiome studies have reported high diversity and large variation across samples for species at abundances<1% [68,69]. Therefore, to more accurately identify the raw milk core community, a threshold of ≥1.5% in at least one raw milk control sample, along with the presence in all raw milk controls, was applied. Following the application of these two criteria, 17 genera and 28 species were detected as part of the core raw milk microbiota (Tables S3 and S2). The genera *Lactococcus*, *Pseudomonas* and *Acinetobacter* were most abundant, ranging from 0.48 to 68.77%, 0.002 to 79.95% and 0.15 to 32.82%, respectively. Within the *Lactococcus* genus, three species, *Lactococcus lactis*, *Lactococcus cremoris* and *Lactococcus raffinolactis*, were detected, with their abundances spanning 0.07–35.34%, 0.11–37.49% and 0.03–1.68%, respectively. Although *L. lactis* did not reach the highest maximum abundance per sample, it was often present in the raw milk controls at relatively high abundances exceeding 20%. The abundance of *L. cremoris* showed more pronounced fluctuations, while that of *L. raffinolactis* remained consistently low in the raw milk controls. The species *Pseudomonas lundensis* constituted the majority of the representatives of the *Pseudomonas* genus detected, with its relative abundance reaching a maximum of 79.89% (in F3), ranging as low as 0.002–5.23% in others, indicating significant variations in its (relative) abundance across the raw milk controls. The top two species within the *Acinetobacter* genus were *Acinetobacter johnsonii* and *Acinetobacter guillouiae*, with abundances ranging from 0.002 to 23.97% and 0.01 to 13.74%, respectively.

**Fig. 3.**
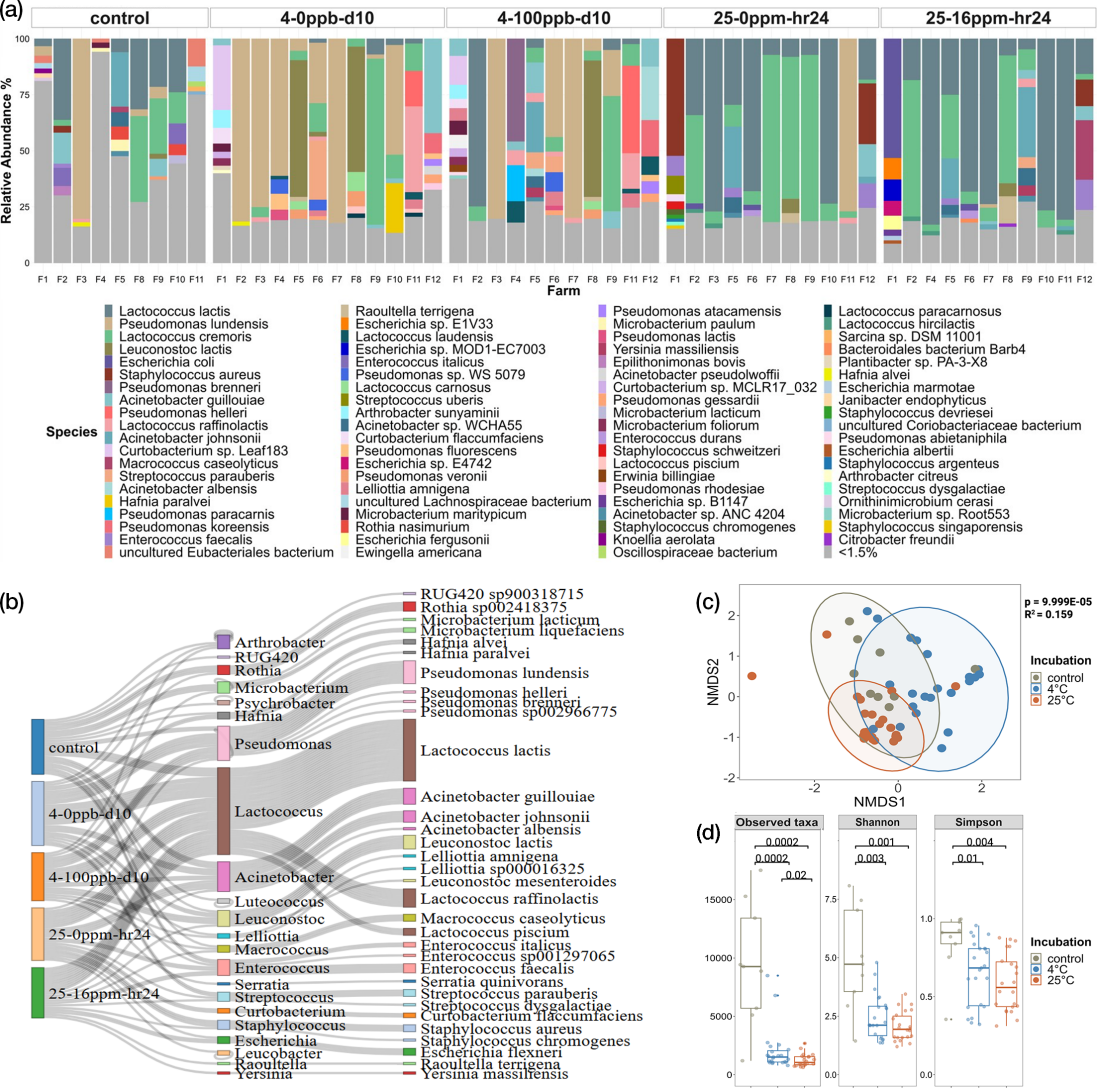
Taxonomic profiles of raw milk controls and incubated samples. (**a**) Bar plot illustrating taxonomic profiles at species level as relative abundance percentages (*y*-axis) obtained from short-read data, facetted in order of raw milk controls (*n*=9), chlorate-unspiked samples from day 10 of 4 °C incubation (*n*=12), chlorate-spiked samples from day 10 of 4 °C incubation (*n*=11), chlorate-unspiked samples from hour 24 of 25 °C incubation (*n*=11) and chlorate-spiked samples from hour 24 of 25 °C incubation (*n*=11). Species with less than 1.5% relative abundance are grouped as low-abundance community members. MAGs obtained from each incubation condition are shown at both genus and species levels. (**b**) Sankey plot displaying sample source (first column) and taxonomic lineage at genus (middle column) and species (last column) levels for the 118 high-quality MAGs obtained. Beta and alpha diversity analysis across incubation conditions. (**c**) NMDS plot showing beta diversity measurements using Bray–Curtis distance for different incubation conditions, wherein raw milk controls (*n*=9) are presented by grey points, 4 °C samples (*n*=23) by blue and 25 °C samples (*n*=22) by orange points. PERMANOVA test was employed with *R*^2^ and *P*-values (*P*<0.05) displayed to the right of the plot. (**d**) Box plots showing Alpha diversity measurements using the same colour scheme and sample numbers as with beta diversity measurements. *P-*values (*P*<0.05) obtained from the pairwise Wilcox test, followed by Benjamini–Hochberg p-adjustment are reported at the top of each facet. All plots above employed short-read datasets wherein a species-level relative abundance threshold of 0.001% was applied.

Upon incubation, shifts in microbial composition were observed that differed between the 4 and 25 °C incubation conditions. In the 4 °C samples, 4-0ppb-d10 and 4-100ppb-d10, a substantial increase in the abundance of *P. lundensis* was observed compared to the raw milk controls (Fig. 3a). Of the 23 spiked and unspiked samples, 56% exhibited high abundances of *P. lundensis*, often exceeding 40%, with the highest abundance reaching 82% in a sample (F7-4-0ppb-d10). Similarly, *L. cremoris* was detected in 56% of the samples; however, in the majority of samples, its relative abundance varied between 4.13 and 12.55%, with only two samples (F9-0ppb-d10 and F9-100ppb-d10) showing relative abundances of 51.08% and 73.59%. *L. lactis* and *L. raffinolactis* were present in 34% of the samples, with abundances ranging from 1.65 to 6.88% and 1.64 to 6.66%, respectively, for most samples. However, one sample reported a high relative abundance of *L. lactis* at 74.31% (F2-4-100ppb-d10), while two samples showed *L. raffinolactis* relative abundances of 15.69% and 37.79% (both from F11). *A. guillouiae* also showed increased relative abundance in the 4 °C samples compared to the raw milk controls, being present in 39% of the samples with relative abundances ranging from 1.77 to 13.56%, except for one sample (F12-4-0ppb-d10) which reached 41.13%. *Leuconostoc lactis*, although present in only 21% of the samples, showed a substantial increase in relative abundance in 4 °C samples compared to the raw milk controls. When present, *Leu. lactis* exhibited relative abundances greater than 55% in almost all samples (F5-4-0ppb-d10, F8-0ppb-d10, F8-100ppb-d10). Additionally, it was observed that higher relative abundances of *L. cremoris*, *L. lactis*, *L. raffinolactis*, *A. guillouiae* and *Leu. lactis* in a sample were associated with lower relative abundances or complete absence (or at least below detection limit) of *P. lundensis*.

For the 25 °C samples, 25-0ppm-hr24 and 25-16ppm-hr24, *L. lactis* and *L. cremoris* continued to be the most abundant taxa, with their relative abundance per sample increasing upon incubation. *L. lactis* was observed in 86% of the 23 samples incubated at 25 °C, with relative abundances often exceeding 15%, where more than half of these samples had relative abundances greater than 67% (Fig. 3a). *L. cremoris* was present in 91% of the samples, but most exhibited lower relative abundances ranging from 1.65 to 9.5%. Higher relative abundances, ranging from 39 to 74%, were reported in only one-third of the samples with *L. cremoris*. Increased relative abundances were also observed for *A. johnsonii*, which was present in 27% of the samples. The lower relative abundances of *A. johnsonii* ranged from 1.9 to 30%, with about half of the samples closer to the lower end and the other half closer to the higher end of that range. With the 25 °C samples, *Staphylococcus aureus* and *Escherichia coli* showed higher relative abundances compared to the raw milk controls, but were present in only 3 and 4 of the 23 samples, respectively. High relative abundances greater than 50% for *S. aureus* and *E. coli* were reported in only one of the three and one of the four samples, F1-25-0ppm-hr24 and F1-25-16ppm-hr24, respectively. The other samples had lower relative abundances at 11.75% and 26.86% for S. *aureus* and ranging from 2.56 to 3.13% for *E. coli*. Decreased relative abundances were observed for *P. lundensis* after incubation at 25 °C relative to the raw milk controls. *P. lundensis* was present in only 2 of the 23 samples, with a high relative abundance of 76.73% reported for one sample (F11-25-0ppm-hr24) and the other being at a low relative abundance of 1.78%. In samples where increased relative abundances of *S. aureus*, *E. coli* and *P. lundensis* were observed, *L. lactis* was absent (or below detection limit), and *L. cremoris* was either at low relative abundances or absent (or below detection limit) as well.

A total of 118 MAGs were obtained. A large proportion of these belonged to the genera *Lactococcus*, *Pseudomonas* and *Acinetobacter*, reflecting their relative abundance in the short-read taxonomic profiles (Fig. 3b). The MAGs obtained from each of the three genera were as follows: 4, 2 and 3 from raw milk controls; 15, 11 and 5 from 4 °C incubated samples; and 19, 1 and 5 from 25 °C incubated samples, respectively. Within the *Lactococcus* genus, MAGs (numbers indicated in brackets) were obtained for *L. lactis* (27), *L. raffinolactis* (8) and *Lactococcus piscium* (3), which was present in very low relative abundances based on short-read taxonomic profiles. However, no MAGs were obtained for *L. cremoris*. For the *Pseudomonas* genus, MAGs included *P. lundensis* (10), *Pseudomonas helleri* (1), *Pseudomonas brenneri* (1), *P. sp00296675* (1) and an unidentified *Pseudomonas* species (1), with the latter two not identified from the short-read taxonomic profiles. Most MAGs from *Acinetobacter* were from *A. guillouiae* (7), followed by *A. johnsonii* (5) and *Acinetobacter albensis* (1). MAGs from the genus *Leuconostoc* were mostly from *Leu. lactis* (6), along with one from *Leuconostoc mesenteroides*. MAGs were also obtained for the genera *Psychrobacter* (1), *Luteococcus* (1), *Serratia* (1) and *Leucobacter* (1), which were not identified from short-read taxonomic profiles (Figure S4).

Consistent taxonomic profiles were obtained for both spiked and unspiked samples under each incubation condition. The incubation temperature significantly affected the microbiota of the raw milk samples, while the presence of chlorate did not. This is supported by statistical significance determined by PERMANOVA for incubation (*P*=9.999E-05, *R*² = 0.159) (Fig. 3c) and a lack of significance when comparing spiked and unspiked communities under the same incubation temperature (4 °C: *P*=0.872, *R*²=0.024; 25 °C: *P*=0.805, *R²*=0.026). The 4 °C and 25 °C samples differed significantly from each other (*P*=0.003) and from the raw milk controls (*P*=0.029 for both 4 °C and 25 °C) as determined by pairwise PERMANOVA. Significant differences were also observed in the alpha diversity metrics (observed taxa, Shannon, Simpson) based on incubation temperature, rather than the presence of chlorate (Fig. 3d). Greater numbers of taxa were observed in the raw milk controls compared to the 4 °C (*P*=0.0002) and 25 °C (*P*=0.0002) samples, with the lowest number of observed taxa in the 25 °C samples compared to the 4 °C samples (*P*=0.02). The Shannon and Simpson metrics also indicated significantly higher diversity in the raw milk controls than in the 4 °C (Shannon: *P*=0.003; Simpson: *P*=0.01) and 25 °C samples (Shannon: *P*=0.001; Simpson: *P*=0.004).

### Incubation temperature selects for microbial genera that harbour various genes involved in chlorate reduction

Following taxonomic profiling, the functional capabilities of raw milk microbial metagenomes were assessed by determining the presence of genes involved in chlorate reduction. These included perchlorate reductase (*pcrA*), chlorate reductase (*clrA*) and nitrate reductases in the DNR and denitrification pathways (*narG, narH, narI, napA* and *napB*), along with a nitrate reductase in the ANR pathway (*nasA*). Additionally, genes coding for selenate reductases (*ynfE* and *ynfF*), DMSO reductases (*dmsA2* and *dmsA*), TMAO reductases (*torA* and *torZ*), biotin sulfoxide reductase (bisC) and chlorite dismutase (*cld*) were identified. Molybdopterin oxidoreductases (*ydeP* and *unk_mol*) and other members identified as belonging to the chlorite dismutase family (*ywfI*) were also detected. The genes for nitrite reductase from the DNR and denitrification pathways (*nirB, nrfH* and *nirS*), along with those for nitric oxide and nitrous oxide reductases in the denitrification pathway (*norC* and *nosZ*), were also screened. The relevant genes extracted from MAGs were mapped back to the reads to quantify their abundances in the raw milk controls and samples (Table S3). Differences in gene abundances were determined through differential abundance analysis using ALDEx2 (Table S4), with significance thresholds set at *P*<0.05 and FDR<0.05. Spearman correlation analysis was performed to elucidate the relationships between taxa and functional genes within the samples (Table S4), reporting only statistically significant correlations (*P*<0.05).

**Fig. 4.**
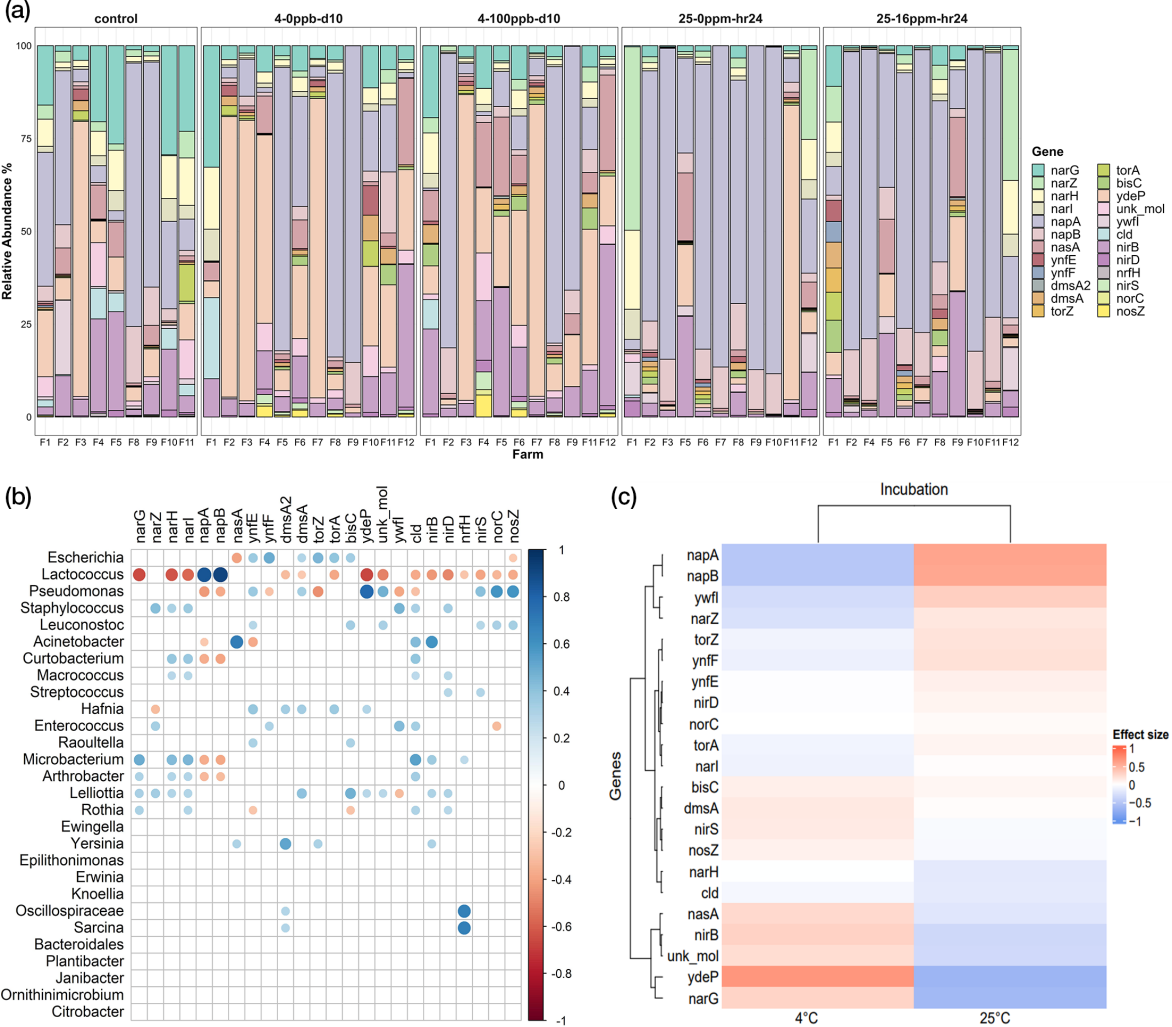
Chlorate reduction functional profiles of raw milk controls and incubated samples. (**a**) Bar plot depicting the relative abundance percentages (*y*-axis) of genes of interest potentially involved in chlorate reduction, alongside those related to DNR and denitrification pathways, for each sample (*x*-axis), facetted in order of: raw milk controls (*n*=9), chlorate-unspiked samples from day 10 of 4 °C incubation (*n*=12), chlorate-spiked samples from day 10 of 4 °C incubation (*n*=11), chlorate-unspiked samples from hour 24 of 25 °C incubation (*n*=11) and chlorate-spiked samples from hour 24 of 25 °C incubation (*n*=11). Spearman correlation analysis of bacterial genera and potential chlorate-reducing genes of interest. (**b**) Heatmap coloured by Spearman correlation coefficient from positive (blue) to negative (red) associations with bubble sizes indicating the number of MAGs for which the correlation coefficient value was observed. Conducted across all raw milk controls and incubated samples together (*n*=54), wherein only statistically significant correlations (*P*<0.05) are displayed. Differential abundance analysis using ALDEx2 for potential chlorate-reducing genes of interest and incubation conditions. (**c**) Heatmap coloured by effect sizes calculated from differential abundance analysis for the genes of interest in 4 °C (*n*=23) and 25 °C (*n*=22) incubated samples, where higher effect size indicates increased abundances (in red) and lower or negative effect sizes indicate reduced abundances (in blue).

Genes directly linked to perchlorate and/or chlorate reduction (*pcrA* and *clrA*) were not detected in either the raw milk controls or incubated samples. Consequently, this section focuses on other genes previously associated with chlorate reduction in the literature. Gene abundances varied significantly across incubation temperature yet did not differ significantly between the spiked and unspiked samples under the same temperature condition, mirroring the pattern observed with respect to taxonomic composition (Fig. 4a). Specifically, the *ydeP* and *narG* genes showed higher abundances in samples incubated at 4 °C compared to both the raw milk controls (*ydeP*, FDR=0.053; *narG*, FDR=0.023) and the 25 °C incubated samples (*ydeP*, FDR=0.002; *narG*, FDR=0.032). Conversely, genes *napA* and *napB* were more abundant in the 25 °C samples compared to the raw milk controls (*napA*, FDR=0.001) and the 4 °C samples (*napA*, FDR=0.016; *napB,* FDR=0.01). The high abundances of the *Pseudomonas* genus are likely a major contributor to the higher abundances of *ydeP* within the 4 °C samples, while the increased abundances of the *Lactococcus* genus in the 25 °C samples appear to be linked to increased levels of the *napA* and *napB* genes (Fig. 4b, c). The positive association of *narG* with the 4 °C samples could be attributed to less dominant genera (2.54–11.06%), including *Lelliottia, Microbacterium* and *Arthrobacter*, rather than the most dominant ones.

Additionally, the association of potential chlorate-reducing gene abundances along with the level of chlorate reduction observed was also investigated. For this, chlorate-spiked samples were grouped based on the varying levels of chlorate reduction observed as high (66%–100%), medium (66%–33%) and low (<33%). Increased *ydeP* gene abundance was observed in high reduction samples, but not at a level of statistical significance. Similarly, higher *napA* gene abundance was seen in medium and low reduction samples, without statistical significance (Figure S5). However, the temperature of incubation was a confounding factor, with high reduction samples mostly belonging to the 4 °C incubation group and medium and low samples belonging to the 25 °C incubation group. Thus, the high abundance of the *ydeP* gene in high reduction samples is likely reflective of the high abundance of *Pseudomonas* in the 4 °C samples, while higher *napA* gene abundance in medium and low reduction samples corresponds to higher abundance of *Lactococcus* in the 25 °C samples.

Genes predicted to belong to the chloride dismutase family (*cld* and *ywfI*) were the most ubiquitously present, found in 8 of the 28 genera: *Staphylococcus*, *Acinetobacter*, *Curtobacterium*, *Macrococcus*, *Enterococcus*, *Microbacterium*, *Arthrobacter* and *Rothia*. The genera, *Pseudomonas*, *Lactococcus* and *Leuconostoc*, among the high abundance ones and *Escherichia*, *Hafnia* and *Raoultella*, among the lower abundant, carried genes potentially involved in chlorate reduction, although within incomplete pathways. Similarly, for the DNR pathway, the genera *Staphylococcus*, *Macrococcus* and *Microbacterium* exhibited incomplete pathways, suggesting cross-feeding. *Lelliota* was the only genus with a strong positive correlation for all DNR genes. Other genera, including *Hafnia*, *Escherichia*, *Raoultella* and *Yersinia*, also displayed complete DNR pathways within MAGs but did not show significant correlations when assigning genes of interest within the short-read datasets, likely due to lower gene abundances in those datasets.

## Discussion

Chlorate consumption has been associated with impaired thyroid function in adults and hindered neurological development in infants and young children [1,4]. Given that milk is one of the largest contributors to an infant’s and young child’s diet, this poses a particular concern for this demographic [5,8]. Therefore, chlorate levels in milk must be maintained below the EU MRL of 0.10 mg kg^−1^ to be considered high quality, in addition to meeting organoleptic and microbiological safety standards. A study by McCarthy *et al*. [22] demonstrated the presence of naturally occurring, chlorate-reducing bacteria in raw milk samples through culture-based methods. In the current study, we further assessed the microbial composition of raw milk samples collected from farms across Ireland through culture-independent shotgun metagenomic sequencing. Specifically, we screened for chlorate-reducing bacteria within these communities and examined the effects of various incubation conditions and chlorate concentrations on them.

Sequencing of raw milk collected from the bulk tanks of nine farms, which served as controls in this study, revealed a core microbiome comprising 17 genera that were consistently present in all raw milk controls and accounted for ≥1.5% relative abundance in at least one sample. A large number of microbial taxa were present at very low abundances, specifically below 1.5%, indicating significant diversity at these low levels [68,71]. Among the genera consistently present, *Lactococcus, Pseudomonas* and *Acinetobacter* dominated much of the community, aligning with findings from other studies on the microbiota of Irish and non-Irish milk [68,69, 71, 72]. In most dairy microbiome studies, including this one, the most abundant species were found to be *L. lactis* and *L. cremoris* within the *Lactococcus* genus, *Pseudomonas lundesis* within the *Pseudomonas* genus and *A. johnsonii* and *A. guillouaea* within the *Acinetobacter* genus [68,70]. The genus *Lactococcus* is recognized as being technologically relevant in the food industry, typically associated with milk rather than the environment, while psychrophilic *Pseudomonas* and *Acinetobacter* spp. are often associated with cold storage and are linked to environmental sources [68,73,77]. Upon incubation at 4 °C, the relative abundance of *Pseudomonas* and *Acinetobacter* increased, reflecting the microbiome shifts typically seen during bulk tank storage of raw milk in winter months [69,71]. Conversely, an increase in *Lactococcus* was observed at 25 °C, mirroring the microbiome changes generally seen during bulk tank storage of raw milk in warmer months [69,78]. The addition of chlorate did not significantly influence the microbial community or composition of the raw milk samples. This phenomenon has been observed for raw milk samples, where chlorinated-detergent usage on the farm has not significantly altered the raw milk microbiome [79,80]. However, the lack of impact contrasts with our expectations, as large amounts of chlorate were deliberately added during testing, compared to the relatively low levels that might be introduced inadvertently through farm cleaning practices. Despite the addition of higher chlorate concentrations in our study, the presence of chlorate did not affect microbial growth or alter taxonomic and functional profiles under the conditions tested in this work, while incubation temperature had a significant impact.

Differential abundance analysis revealed an increase in the *narG* and *ydeP* genes within the 4 °C samples, of which *narG* is known to be involved in chlorate reduction [21,33, 35,39]. Additionally, both *narG* and *narZ* genes have been reported to be closely related to the *pcrA* gene [29,81, 82]. *narG* was associated with lower relative abundance genera in the 4 °C samples, including *Lelliottia*, *Microbacterium* and *Arthrobacter*, indicating that less dominant community members played a role in chlorate reduction. *Pseudomonas* was observed to be the most abundant in 4 °C samples, showing a strong positive association with the *ydeP* gene. However, while high levels of chlorate reduction were observed in *Pseudomonas*-dominated samples, the role of *ydeP* in chlorate reduction is not yet fully understood. Therefore, the precise contribution of *Pseudomonas* genus members to chlorate reduction could not be determined. In this study and in previous reports, *ydeP* has been identified as similar to genes encoding formate dehydrogenase [35]. Although some reports mention the structural similarity between formate and chlorate, suggesting potential chlorate reduction by formate dehydrogenase, the precise mechanism remains unclear [33,71, 83]. In the 25 °C incubated samples, increased abundances of the periplasmic nitrate reductase genes, *napA* and *napB*, were observed, which were strongly positively correlated with the most abundant genus in these samples, *Lactococcus*. Previous reports have linked *napA* to chlorate reduction, albeit to much lower rates than that of *narG* [33,36,38, 84]. Similarly, in this study, the 25 °C chlorate-spiked samples with increased abundances of the *napA* gene exhibited low to medium (up to 66%) levels of chlorate reduction, compared to the 4 °C chlorate-spiked samples with increased abundances of *narG* that showed high (greater than 66%) levels of chlorate reduction. However, given the high chlorate concentration (16 ppm) added to the 25 °C samples, even low to medium reduction levels (up to 66%) reflect a considerably large extent of chlorate removal.

In this study, genes belonging to the chlorite dismutase family, *cld* and *ywfI*, were ubiquitously present, being identified in 8 out of 28 genera. The enzyme Cld is responsible for breaking down toxic chlorite into chloride ions and molecular oxygen [21,35, 85]. Similar to this study, previous reports have also suggested a widespread presence of genes encoding *cld* to protect bacterial community members from chlorite toxicity produced through co-metabolism of chlorate or through non-microbially mediated metabolism of chlorate in the environment [86,88]. Genes coding for Cld have also been reported to show high diversity, explained by the frequent occurrence of horizontal gene transfer events of these genes as part of perchlorate reduction genomic islands [89,90]. However, the *cld* family is also susceptible to misannotations due to highly similar sequences within the family for which functions are unknown [86,91, 92]. Therefore, distinguishing true diversity of the *cld* genes from misannotated *cld* genes is currently a difficult task.

Additionally, when examining the presence of genes involved in chlorate-reducing and chlorite dismutation across various genera, the presence of incomplete pathways for chlorate reduction and removal was observed. The genera *Pseudomonas*, *Lactococcus*, *Leuconostoc*, *Escherichia*, *Hafnia* and *Raoultella* possessed genes involved in chlorate reduction but lacked those involved in chlorite dismutation. Conversely, genes involved in chlorite dismutation were identified in genera that lacked other genes for chlorate reduction, indicating the possibility of metabolic cross-feeding among microbial community members involved in chlorate reduction and chlorite dismutation. Similar interactions have been reported for known DPRB [93]. However, understanding these metabolic cross-interactions in microbial communities is complex due to the numerous genes and potential pathways involved in chlorate reduction, the unknown functions of many genes within the DMSO family predicted to be involved in chlorate reduction [35,93] and the lack of these pathways being annotated in routine pathway mapping databases such as the Kyoto Encylopedia of Genes and Genomes (KEGG) [21]. Improvements in these areas will enhance the accuracy of gene prediction in these communities, allowing metagenomic studies to provide deeper insights into microbial interactions involved in chlorate reduction.

Overall, this study identifies key bacterial species within the raw milk microbiome that harbour genes predicted to be involved in chlorate reduction, highlighting an under-explored functional potential in the dairy context. While chlorate-reducing bacteria have previously been identified in chlorate-contaminated sites, such as soil and wastewater systems, and studied for their application in the bioremediation of such sites [19,21,94], their presence and role in raw milk remain poorly understood. This work provides a foundational step toward detecting chlorate reduction genes in raw milk bacterial genomes, presenting these bacteria as candidates for further investigation in chlorate mitigation within the dairy industry. However, as a metagenomic study, this work is exploratory, and further meta-transcriptomic and proteomic studies will be necessary to confirm gene expression and clarify the underlying mechanisms. Furthermore, the role of inter-kingdom interactions between fungal species and chlorate-reducing bacteria remains largely unexplored across sample sources, including raw milk, and studying these interactions could further elucidate cross-community processes involved in chlorate reduction.

## Conclusions

This study demonstrates that raw milk naturally harbours microbes capable of reducing chlorate. Incubation temperature significantly influenced the raw milk microbiome more than the presence of chlorate at the various concentrations tested. While no genes directly linked to perchlorate or chlorate reductase were observed, genes associated with nitrate reduction, such as the nitrate reductases *narG* and *napA*, which have been reported to be involved in chlorate reduction, were present in chlorate-spiked milk samples. Incomplete pathways for chlorate reduction were observed in many genera, suggesting potential metabolic cross-feeding mechanisms. Decoding these community interactions is currently hindered by several factors, the most prominent being the presence of many genes predicted to be involved in chlorate reduction but lacking characterized functions. Future efforts should focus on the biochemical characterization of these genes, enabling metagenomic studies to understand their roles within microbial communities, thereby providing valuable information for the development of effective chlorate mitigation strategies.

## Supplementary material

10.1099/acmi.0.001088.v3 Uncited Supplementary Material 1.

10.1099/acmi.0.001088.v3 Uncited Supplementary Material 2.

## Abbreviations

Cld: chlorite dismutase
ClrA: chlorate reductase
DMSO: dimethyl sulfoxide
DNR: dissimilatory nitrate reduction
DPRB: dissimilatory (per)chlorate-reducing bacteria
EU: European Union
FDR: false discovery rate
gDNA: genomic DNA
GTDB: Genome Taxonomy Database
KEGG: Kyoto Encylopedia of Genes and Genomes
MAG: metagenome-assembled genomes
MRL: maximum residue limit
PcrA: perchlorate reductase
ppb: parts per billion
ppm: parts per million
TMAO: trimethylamine-N-oxide
