# Supplementary material for: Investigating the role of bacterial raw milk community members in chlorate reduction
Source: Access Microbiol. 2026 Apr 20;8(4):001088.v3. doi: 10.1099/acmi.0.001088.v3 (PMC13094775; doi:10.1099/acmi.0.001088.v3)
Supplement: Uncited Supplementary Material 1. [file acmi-8-01088-s001.pdf]

Supplementary Material

Supplementary Tables

Supplementary Table 1. Range of chlorate concentrations tested during initial screening to determine the maximum level at which chlorate reduction was observed in the 25°C incubated samples. Initial chlorate concentrations are shown at hour 0 and final concentrations at hour 24 for each replicate.

Chlorate concentration added	Chlorate concentration in replicate 1		Chlorate concentration in replicate 2	
	hour 0	hour 24	hour 0	hour 24
0 ppm	<2.0	<2.0	<2.0	<2.0
0.25 ppm	0.05	0.03	0.26	0.19
0.5 ppm	0.50	0.51	0.52	0.45
1 ppm	1.00	0.79	1.09	0.62
2 ppm	2.00	2.00	2.19	2.11
4 ppm	4.30	4.30	4.24	4.23
8 ppm	9.20	2.60	9.05	8.42
16 ppm	16.60	8.60	16.34	10.98
32 ppm	33.50	31.90	32.05	28.93
64 ppm	59.00	65.00	64.93	62.21
120 ppm	121.00	115.00	121.57	119.69

Supplementary Table 2. Number of reads obtained per sample and reads lost after host removal.

Sample	Reads per sample (in millions)	Reads post host removal (in millions)	Reads lost during host removal (%)
F1-control	10.7	4.5	57.9
F1-4-0ppb-d10	3.4	2.6	23.5
F1-4-100ppb-d10	10.1	8.5	15.8
F1-25-0ppm-hr24	84.5	64.1	24.1
F1-25-16ppm-hr24	23.8	23.6	0.8
F2-control	4.2	3.2	23.8
F2-4-0ppb-d10	8.5	8.5	0.0
F2-4-100ppb-d10	10.6	10.5	0.9
F2-25-0ppm-hr24	3.1	3.1	0.0
F2-25-16ppm-hr24	3.3	3.3	0.0
F3-control	16.5	16.5	0.0
F3-4-0ppb-d10	7.4	7.4	0.0
F3-4-100ppb-d10	5.7	5.7	0.0
F3-25-0ppm-hr24	26.3	25.7	2.3
F4-control	83.1	56.9	31.5
F4-4-0ppb-d10	101.0	100.9	0.1
F4-4-100ppb-d10	2.4	2.4	0.0
F4-25-16ppm-hr24	7.0	6.9	1.4
F5-control	25.4	14.2	44.1
F5-4-0ppb-d10	1.7	1.7	0.0
F5-4-100ppb-d10	2.0	2.0	0.0
F5-25-0ppm-hr24	9.2	9.1	1.1
F5-25-16ppm-hr24	4.2	4.2	0.0
F6-4-0ppb-d10	2.2	2.2	0.0
F6-4-100ppb-d10	47.6	47.1	1.1
F6-25-0ppm-hr24	4.5	4.5	0.0
F6-25-16ppm-hr24	4.5	4.4	2.2

F7-4-0ppb-d10	1.8	1.8	0.0
F7-4-100ppb-d10	4.3	4.3	0.0
F7-25-0ppm-hr24	15.5	15.4	0.6
F7-25-16ppm-hr24	5.5	5.5	0.0
F8-control	14.4	11.7	18.8
F8-4-0ppb-d10	3.8	3.8	0.0
F8-4-100ppb-d10	1.9	1.9	0.0
F8-25-0ppm-hr24	4.0	4.0	0.0
F8-25-16ppm-hr24	3.0	3.0	0.0
F9-control	218.6	26.7	87.8
F9-4-0ppb-d10	12.1	12.0	0.8
F9-4-100ppb-d10	498.5	495.9	0.5
F9-25-0ppm-hr24	12.6	12.6	0.0
F9-25-16ppm-hr24	4.1	4.0	2.4
F10-control	19.7	18.0	8.6
F10-4-0ppb-d10	9.5	9.5	0.0
F10-25-0ppm-hr24	7.9	7.9	0.0
F10-25-16ppm-hr24	9.3	9.3	0.0
F11-control	240.5	149.2	38.0
F11-4-0ppb-d10	3.7	3.7	0.0
F11-4-100ppb-d10	1.2	1.1	8.3
F11-25-0ppm-hr24	4.0	3.9	2.5
F11-25-16ppm-hr24	2.9	2.9	0.0
F12-4-0ppb-d10	80.2	78.2	2.5
F12-4-100ppb-d10	8.9	8.7	2.2
F12-25-0ppm-hr24	4.0	3.9	2.5
F12-25-16ppm-hr24	2.6	2.5	3.8

Supplementary Table 3. Genera identified from short-read taxonomic profiles for the raw milk controls (3a) and incubation conditions (3b & 3c), along with the relative abundance (%) of each genus per farm.

3a) Raw milk controls

**Genus**	**F1**	**F2**	**F3**	**F4**	**F5**	**F8**	**F9**	**F10**	**F11**
Lactococcus	3.93	38.25	1.89	0.58	6.62	68.77	45.86	37.40	0.48
Pseudomonas	4.00	0.86	79.95	0.05	0.00	4.07	6.93	1.23	0.02
Acinetobacter	0.15	13.80	0.95	2.02	32.82	0.68	7.96	1.25	0.03
uncultured	6.23	0.41	0.02	2.71	2.07	1.33	2.31	2.55	20.34
Enterococcus	0.06	9.59	0.01	0.09	0.43	0.11	0.14	9.24	0.03
Microbacterium	0.02	1.60	0.00	4.58	5.81	0.03	0.08	4.31	0.03
Rothia	0.01	0.03	0.00	0.07	5.92	0.00	0.00	5.02	0.02
Epilithonimonas	0.00	4.27	0.05	0.12	0.00	0.00	0.00	0.00	0.00
Oscillospiraceae	0.57	0.04	0.00	0.22	0.18	0.12	0.21	0.31	2.23
Knoellia	2.25	0.04	0.00	1.29	0.09	0.03	0.03	0.02	0.06
Janibacter	1.80	0.02	0.00	0.98	0.05	0.01	0.02	0.21	0.49
Sarcina	0.43	0.02	0.00	0.19	0.13	0.08	0.11	0.30	2.06
Staphylococcus	0.01	2.96	0.00	0.02	0.10	0.00	0.00	0.00	0.09
Ornithinimicrobium	1.58	0.03	0.00	0.98	0.07	0.02	0.06	0.18	0.18
Leuconostoc	0.03	0.02	0.01	0.00	0.01	0.30	2.37	0.33	0.00
Macrococcus	0.00	0.00	0.00	0.00	2.23	0.32	0.16	0.18	0.01
Hafnia	0.03	0.24	1.89	0.00	0.00	0.01	0.01	0.00	0.00

3b) 4°C incubated samples

**Unspiked**	**F1**	**F2**	**F3**	**F4**	**F5**	**F6**	**F7**	**F8**	**F9**	**F10**	**F11**	**F12**
Pseudomonas	0.00	81.31	74.81	79.04	9.74	33.58	82.01	6.57	2.04	48.95	15.79	20.92
Lactococcus	0.00	0.00	6.57	1.54	7.86	15.84	0.00	17.42	80.47	13.04	58.61	0.00
Leuconostoc	0.00	0.00	0.00	0.00	60.77	2.25	0.00	55.71	0.00	0.00	0.00	0.00
Acinetobacter	2.96	0.00	0.00	0.00	0.00	0.00	0.00	0.00	1.78	2.11	0.00	45.09
Curtobacterium	38.33	0.00	0.00	0.00	0.00	0.00	0.00	0.00	0.00	0.00	0.00	0.00

Streptococcus	0.00	0.00	0.00	0.00	0.00	26.17	0.00	0.00	0.00	0.00	0.00	0.00
Lelliottia	0.00	0.00	0.00	0.00	0.00	2.54	0.00	0.00	0.00	0.00	4.12	0.00
Hafnia	0.00	1.84	0.00	0.00	0.00	0.00	0.00	0.00	0.00	22.23	0.00	0.00
Microbacterium	6.48	0.00	0.00	0.00	0.00	0.00	0.00	0.00	0.00	0.00	0.00	0.00
Arthrobacter	9.48	0.00	0.00	0.00	0.00	0.00	0.00	0.00	0.00	0.00	0.00	0.00
Ewingella	0.00	0.00	0.00	0.00	0.00	0.00	0.00	0.00	0.00	0.00	0.00	0.00
Yersinia	0.00	0.00	0.00	0.00	0.00	0.00	0.00	0.00	0.00	0.00	0.00	0.00
Erwinia	0.00	0.00	0.00	0.00	0.00	0.00	0.00	0.00	0.00	0.00	0.00	0.00
Raoultella	0.00	0.00	0.00	0.00	1.75	0.00	0.00	0.00	0.00	0.00	0.00	0.00
Plantibacter	1.89	0.00	0.00	0.00	0.00	0.00	0.00	0.00	0.00	0.00	0.00	0.00
**Spiked-100ppb**	**F1**	**F2**	**F3**	**F4**	**F5**	**F6**	**F7**	**F8**	**F9**	**F11**	**F12**	
Pseudomonas	1.66	0.00	80.03	61.45	0.00	56.59	79.52	9.73	20.61	38.79	28.01	
Lactococcus	0.00	80.98	0.00	16.16	14.34	8.42	2.41	7.79	55.94	29.33	8.14	
Leuconostoc	0.00	0.00	0.00	0.00	0.00	0.00	0.00	60.83	0.00	0.00	0.00	
Acinetobacter	7.42	0.00	0.00	4.05	45.83	0.00	0.00	0.00	7.64	0.00	35.99	
Curtobacterium	20.99	0.00	0.00	0.00	0.00	0.00	0.00	0.00	0.00	0.00	0.00	
Streptococcus	0.00	0.00	0.00	0.00	7.28	6.99	0.00	0.00	0.00	0.00	0.00	
Lelliottia	5.71	0.00	0.00	0.00	0.00	6.24	0.00	0.00	0.00	6.49	0.00	
Hafnia	0.00	0.00	0.00	0.00	0.00	0.00	0.00	0.00	0.00	0.00	0.00	
Microbacterium	11.06	0.00	0.00	0.00	0.00	0.00	0.00	0.00	0.00	0.00	0.00	
Arthrobacter	5.96	0.00	0.00	0.00	0.00	0.00	0.00	0.00	0.00	0.00	0.00	
Ewingella	5.76	0.00	0.00	0.00	0.00	0.00	0.00	0.00	0.00	0.00	0.00	
Yersinia	0.00	0.00	0.00	0.00	4.11	0.00	0.00	0.00	0.00	0.00	0.00	
Erwinia	3.12	0.00	0.00	0.00	0.00	0.00	0.00	0.00	0.00	0.00	0.00	
Raoultella	0.00	0.00	0.00	0.00	0.00	0.00	0.00	1.73	0.00	0.00	0.00	
Plantibacter	0.00	0.00	0.00	0.00	0.00	0.00	0.00	0.00	0.00	0.00	0.00	

3c) 25°C incubated samples

**Unspiked**	**F1**	**F2**	**F3**	**F5**	**F6**	**F7**	**F8**	**F9**	**F10**	**F11**	**F12**
Lactococcus	0.00	74.61	84.29	38.63	73.66	81.53	70.94	81.11	81.03	5.38	19.63
Acinetobacter	0.00	0.00	0.00	36.31	0.00	0.00	0.00	0.00	0.00	0.00	17.38
Staphylococcus	61.35	0.00	0.00	0.00	0.00	0.00	0.00	0.00	0.00	0.00	26.87
Escherichia	0.00	2.65	0.00	0.00	2.57	0.00	0.00	0.00	0.00	0.00	0.00
Pseudomonas	0.00	0.00	0.00	0.00	0.00	0.00	0.00	0.00	0.00	76.73	0.00
Enterococcus	8.69	0.00	0.00	4.18	2.59	0.00	0.00	0.00	0.00	0.00	11.00
Macrococcus	0.00	0.00	0.00	0.00	0.00	0.00	0.00	0.00	0.00	0.00	0.00
Raoultella	0.00	0.00	0.00	0.00	0.00	0.00	4.56	0.00	0.00	0.00	0.00
Streptococcus	9.82	0.00	0.00	0.00	0.00	0.00	0.00	0.00	0.00	0.00	0.00
Leuconostoc	0.00	0.00	0.00	0.00	0.00	0.00	6.34	0.00	0.00	0.00	0.00
Yersinia	0.00	0.00	0.00	0.00	0.00	0.00	0.00	0.00	0.00	0.00	0.00
Curtobacterium	3.13	0.00	0.00	0.00	0.00	0.00	0.00	0.00	0.00	0.00	0.00
Bacteroidales	0.00	0.00	0.00	0.00	0.00	0.00	0.00	0.00	0.00	0.00	0.00
Citrobacter	0.00	0.00	0.00	0.00	0.00	0.00	0.00	0.00	0.00	0.00	0.00
**Spiked-16ppm**	**F1**	**F2**	**F4**	**F5**	**F6**	**F7**	**F8**	**F9**	**F10**	**F11**	**F12**
Lactococcus	0.00	80.98	87.43	52.78	73.24	79.07	64.07	17.31	84.01	85.19	17.96
Acinetobacter	0.00	0.00	0.00	23.54	0.00	3.87	0.00	45.10	0.00	1.94	6.42
Staphylococcus	0.00	0.00	0.00	0.00	0.00	0.00	0.00	0.00	0.00	0.00	11.75
Escherichia	91.29	0.00	0.00	0.00	3.13	0.00	0.00	0.00	0.00	0.00	0.00
Pseudomonas	0.00	0.00	0.00	0.00	0.00	1.78	0.00	0.00	0.00	0.00	0.00
Enterococcus	0.00	0.00	0.00	2.87	3.37	0.00	0.00	0.00	0.00	0.00	13.54
Macrococcus	0.00	0.00	0.00	0.00	0.00	0.00	0.00	0.00	0.00	0.00	26.18
Raoultella	0.00	0.00	0.00	0.00	0.00	0.00	12.24	0.00	0.00	0.00	0.00
Streptococcus	0.00	0.00	0.00	0.00	0.00	0.00	0.00	4.98	0.00	0.00	0.00
Leuconostoc	0.00	0.00	0.00	0.00	0.00	0.00	5.76	0.00	0.00	0.00	0.00
Yersinia	0.00	0.00	0.00	0.00	0.00	0.00	0.00	4.31	0.00	0.00	0.00
Curtobacterium	0.00	0.00	0.00	0.00	0.00	0.00	0.00	0.00	0.00	0.00	0.00
Bacteroidales	0.00	0.00	0.00	0.00	1.99	0.00	0.00	0.00	0.00	0.00	0.00

Citrobacter	0.00	0.00	0.00	0.00	0.00	0.00	1.51	0.00	0.00	0.00	0.00

Supplementary Table 4. ALDEx2 differential abundance analysis results for genes of interest across various incubation conditions (4a&4b), with Benjamini Hochberg (BH) method used for p-value adjustment for False Discovery Rate (FDR) control. Statistically significant p-values and FDR are indicated with "*" mark ($p < 0.05$, FDR < 0.05).

4a) raw milk controls versus each of the 4°C and 25°C incubated samples

Gene of interest	Controls p-value	Controls p-value.BH	4°C p-value	4°C p-value.BH	4°C Effect size	25°C p-value	25°C p-value.BH	25°C Effect size
cld	0.576	1.000	0.217	0.915	-0.075	0.158	0.795	-0.187
dmsA	0.178	0.614	0.289	0.813	0.149	0.333	0.870	0.022
napA	0.000*	0.001*	0.357	1.000	-0.513	0.117	0.972	0.604
napB	0.178	0.773	0.488	1.000	-0.511	0.115	0.819	0.585
narG	0.001*	0.023*	0.327	0.992	0.290	0.006	0.112	-0.648
narH	0.015	0.206	0.221	0.943	-0.004	0.087	0.742	-0.199
narI	0.546	0.993	0.422	0.976	-0.118	0.499	0.994	0.017
narZ	0.451	0.985	0.237	0.940	-0.273	0.614	1.000	0.164
nasA	0.496	0.967	0.338	0.960	0.253	0.575	0.995	-0.223
nirB	0.000*	0.003*	0.647	1.000	0.300	0.222	0.942	-0.351
nirS	0.104	0.487	0.372	0.924	0.138	0.448	0.941	-0.048
nosZ	0.141	0.551	0.426	0.933	0.092	0.453	0.968	-0.052
torA	0.401	0.926	0.520	0.982	-0.099	0.553	1.000	0.073
unk_mol	0.513	0.991	0.617	1.000	0.230	0.254	0.868	-0.340
ydeP	0.004*	0.053	0.131	0.927	0.693	0.176	0.897	-0.682
ynfE	0.234	0.722	0.488	0.984	-0.016	0.426	0.960	0.104
ynfF	0.120	0.540	0.527	0.986	-0.130	0.401	0.927	0.203
ywfI	0.400	0.905	0.452	0.981	-0.317	0.452	0.964	0.323
bisC	0.091	0.504	0.260	0.863	0.109	0.284	0.853	0.063
nirD	0.250	0.775	0.509	0.989	-0.014	0.505	0.973	0.074
torZ	0.122	0.561	0.525	0.973	-0.106	0.396	0.920	0.189
norC	0.099	0.541	0.518	0.974	0.001	0.476	0.951	0.034

4b) between the 4°C and 25°C incubated samples

Gene of interest	25°C rab.win	4°C rab.win	Effect size	p-value	p-value.BH
bisC	-0.018	0.345	0.109	0.499	0.646
cld	-1.226	-1.200	0.025	0.599	0.749
dmsA	0.302	0.436	0.099	0.514	0.657
napA	6.804	3.487	-0.639	0.003*	0.016*
napB	4.105	1.115	-0.684	0.001*	0.010*
narG	0.965	2.609	0.518	0.007*	0.032*
narH	0.859	1.479	0.147	0.424	0.588
narI	-0.012	-0.164	-0.116	0.552	0.668
narZ	0.566	-0.207	-0.193	0.370	0.552
nasA	1.247	2.138	0.193	0.265	0.442
nirB	2.582	3.462	0.374	0.060	0.171
nirD	*-0.178*	*-0.744*	*-0.142*	*0.477*	*0.628*
nirS	*-1.179*	*-0.840*	*0.043*	*0.510*	*0.660*
norC	*-1.240*	*-1.261*	*-0.055*	*0.615*	*0.742*
nosZ	*-1.307*	*-0.772*	*0.125*	*0.556*	*0.683*
torA	*-0.204*	*-0.874*	*-0.052*	*0.474*	*0.615*

torZ	*-0.348*	*-1.153*	*-0.142*	*0.394*	*0.549*
unk_mol	*-0.399*	*1.156*	*0.363*	*0.122*	*0.244*
ydeP	*1.650*	*4.571*	*0.904*	*0.000**	*0.002**
ynfE	*-0.337*	*-0.946*	*-0.131*	*0.402*	*0.575*
ynfF	*-0.644*	*-1.637*	*-0.231*	*0.401*	*0.494*
ywfI	*0.245*	*-1.588*	*-0.408*	*0.092*	*0.198*

<u>**Supplementary Figures**</u>

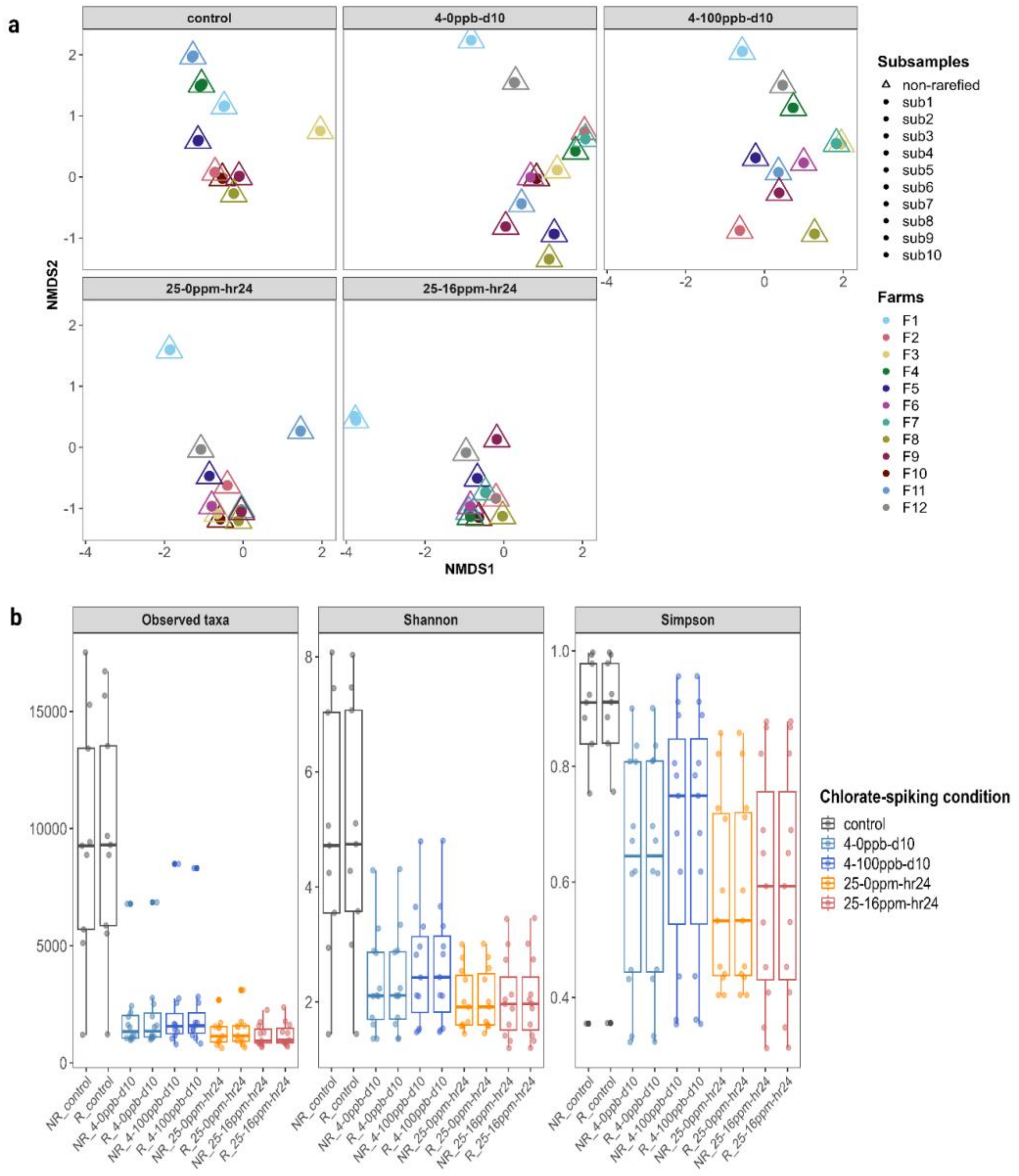

Supplementary Figure 1. (a) and (b) display comparisons of alpha and beta diversity metrics, respectively, among ten subsamples from the short-read datasets rarefied (R) to 1 million reads versus the non-rarefied (NR) datasets. No significant differences were observed in either metric. Both alpha and beta diversity were assessed using a relative abundance threshold of 0.001% at the species level.

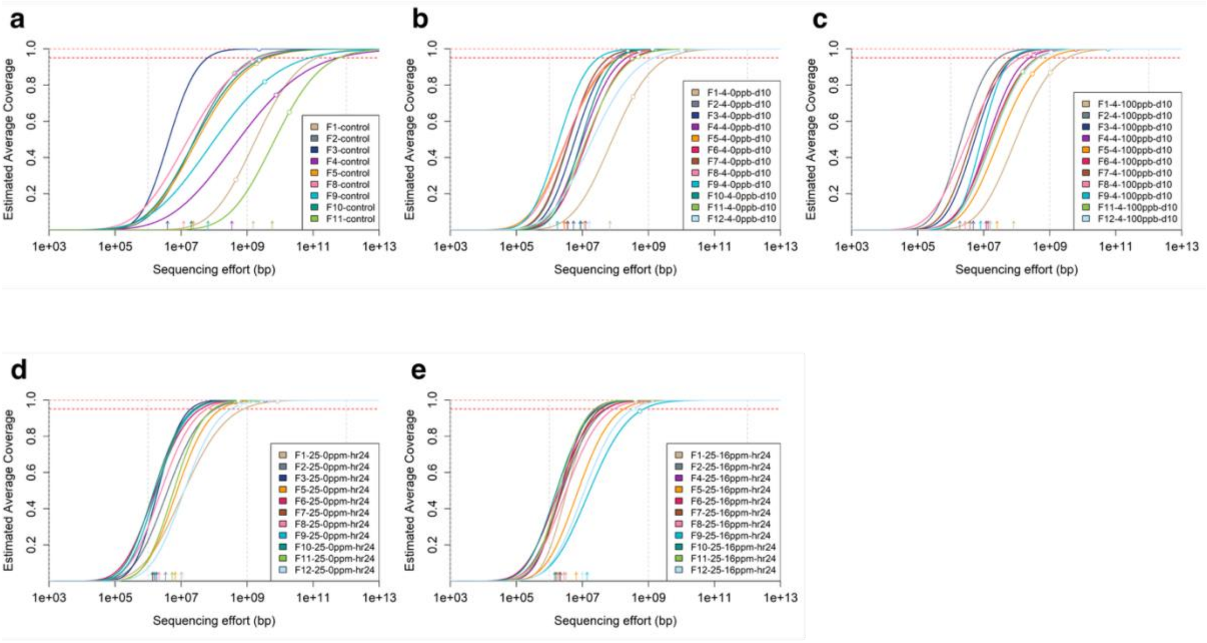

Supplementary Figure 2. Nonpareil curves showing sequencing coverage (y-axis) against sequencing effort in bp (x-axis). The circles represent the actual sequencing coverage achieved for each sample and predicted coverage beyond them with increased sequencing effort for the conditions: (a) raw milk controls, (b) 4-0ppb-d10, (c) 4-100ppb-d10, (d) 25-0ppm-hr24, and (e) 25-16ppm-hr24. High sequencing coverage was generally observed across most samples. However, lower coverage in the raw milk controls was noted, corresponding to samples with lower bacterial counts, as determined by microbial growth analysis, and a high number of host reads identified in the sequencing datasets.

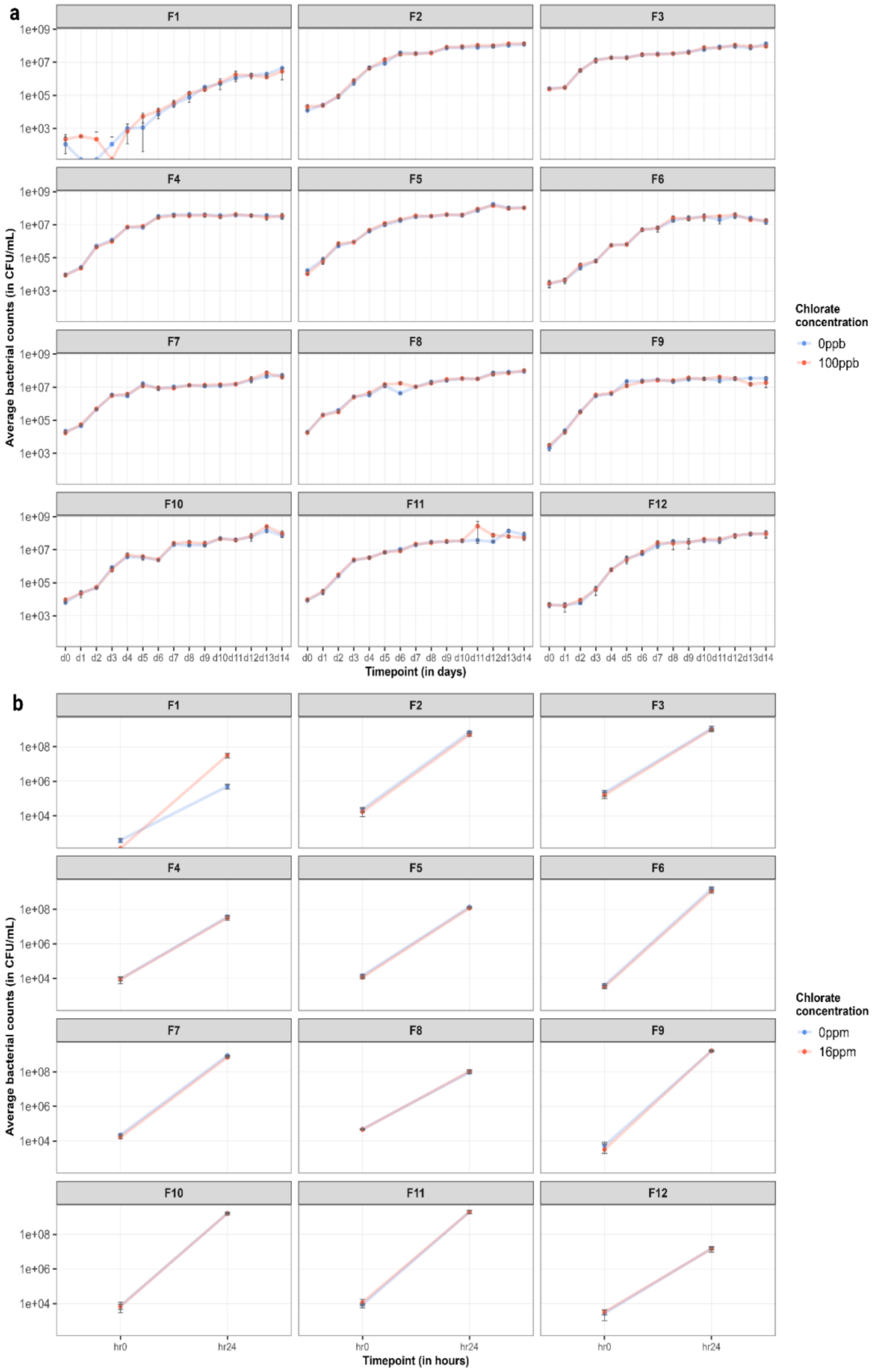

Supplementary Figure 3. (a) and (b) Microbial growth curves of samples incubated at 4°C and 25°C (), respectively, displaying the increase in bacterial load (y-axis) over time (x-axis) in both chlorate-spiked and unspiked samples, for the twelve farms.

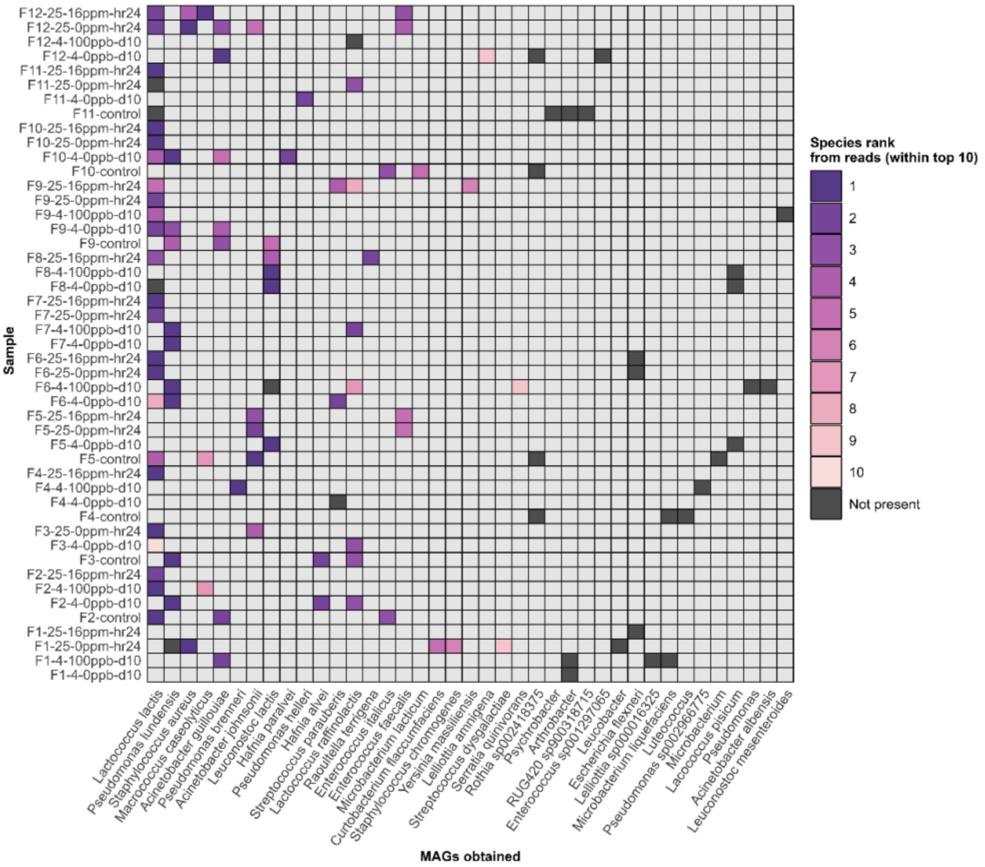

Supplementary Figure 4. Heatmap illustrating the top 10 most abundant taxa identified from short-read taxonomic profiles, along with the corresponding MAGs obtained for these taxa (x-axis) across various samples (y-axis). MAGs were identified for taxa with short-read abundances of ≥ 1.5%. Additionally, some MAGs were obtained for taxa not detected in the short-read taxonomic profiles, indicated in dark grey and labelled as "Not present."

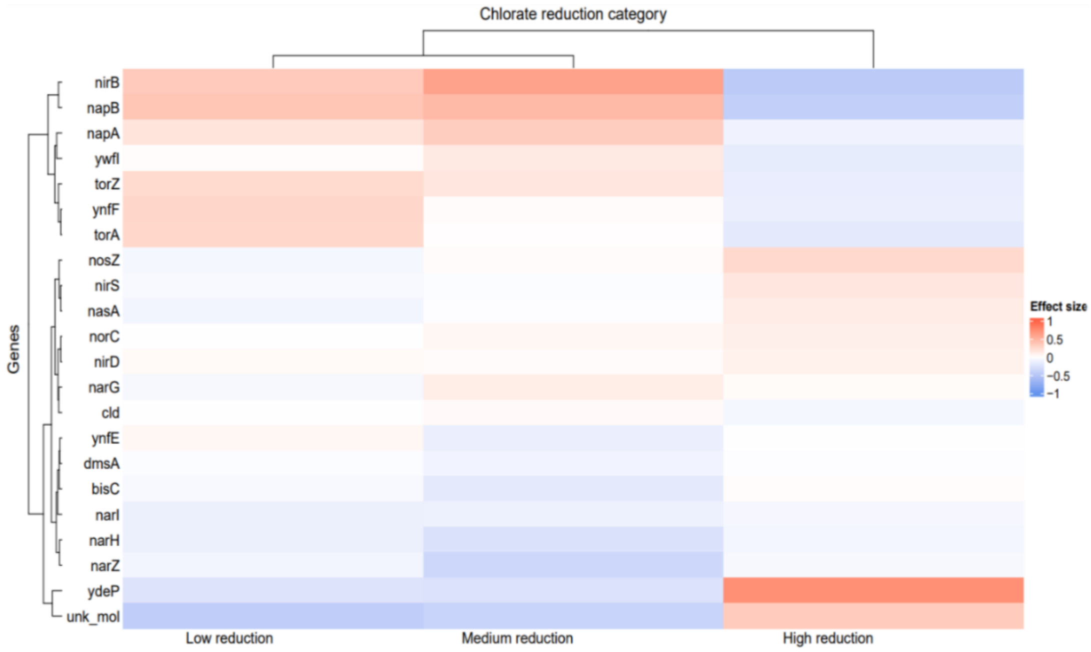

Supplementary Figure 5. Heatmap demonstrating effect sizes from differential abundance analysis for the genes of interest across the three chlorate reduction categories being, high (>66%), medium (33%-66%) and low (<33%), where higher effect size indicates increased abundances (in red) and lower or negative effect sizes indicates reduced abundances (in blue).
